# Comparative genomic mapping of uncharacterized canine retinal ESTs to identify novel candidate genes for hereditary retinal disorders

**Published:** 2009-05-09

**Authors:** B. Zangerl, J. L. Johnson, J. Pillardy, Q. Sun, C. André, F. Galibert, G.M. Acland, G.D. Aguirre

**Affiliations:** 1 Department of Clinical Studies, School of Veterinary Medicine, University of Pennsylvania, Philadelphia, PA; 2 J. A. Baker Institute, College of Veterinary Medicine, Cornell University, Ithaca, NY; 3 Computational Biology Service Unit, Cornell Theory Center, Cornell University, Ithaca, NY; 4 CNRS UMR 6061, Institut de Génétique et Développement de Rennes, Faculté de Médecine, Rennes, France

## Abstract

**Purpose:**

To identify the genomic location of previously uncharacterized canine retina-expressed expressed sequence tags (ESTs), and thus identify potential candidate genes for heritable retinal disorders.

**Methods:**

A set of over 500 retinal canine ESTs were mapped onto the canine genome using the RHDF_5000–2_ radiation hybrid (RH) panel, and the resulting map positions were compared to their respective localization in the CanFam2 assembly of the canine genome sequence.

**Results:**

Unique map positions could be assigned for 99% of the mapped clones, of which only 29% showed significant homology to known RefSeq sequences. A comparison between RH map and sequence assembly indicated some areas of discrepancy. Retinal expressed genes were not concentrated in particular areas of the canine genome, and also were located on the canine Y chromosome (CFAY). Several of the EST clones were located within areas of conserved synteny to human retinal disease loci.

**Conclusions:**

RH mapping of canine retinal ESTs provides insight into the location of potential candidate genes for hereditary retinal disorders, and, by comparison with the assembled canine genome sequence, highlights inconsistencies with the current assembly. Regions of conserved synteny between the canine and the human genomes allow this information to be extrapolated to identify potential positional candidate genes for mapped human retinal disorders. Furthermore, these ESTs can help identify novel or uncharacterized genes of significance for better understanding of retinal morphology, physiology, and pathology.

## Introduction

Heritable disorders of the retina often inflict devastating harm on the lives of affected individuals and their families. Progress in identifying the genetic causes of these diseases has accelerated in recent years, but still presents serious challenges; in many cases nothing is known of the genetic loci involved. Currently, for diseases where the genetic locus has been mapped, close to 30% have not yet had the responsible gene or mutation identified (RetNet). A limiting factor to this progress is an incomplete catalog of the genes expressed in the retina [1].

Animals also suffer from heritable retinal disorders. This is a source of concern when it afflicts companion or working animals and yet affected animals can be a valuable resource when used as models of human disorders. The broadly similar features of retinitis pigmentosa (RP) in human families and progressive retinal atrophy (PRA) in dogs have prompted extensive, productive, and mutually beneficial comparative studies of these homologous disorders [2].

Expressed sequence tags (ESTs) have played an extremely powerful role in the identification and cataloguing of new and tissue-specific genes [3]. The combination of EST discovery with radiation hybrid (RH) mapping has been invaluable to the development, assembly, and annotation of the human gene map [4], a critical first step to an assessment of individual disease loci. However, identification of disease-causing genes within mapped loci that typically have 0.5–10 cM intervals is problematic as the human genome can harbor 5–300 potential candidate genes for a single disorder in these intervals [5]. Comparative genomics is often used to further refine these results. This has recognized limitations if the identification of homolog genes is based only on the alignment of cDNA clones, which may not always cover areas of sufficient conservation between the compared species [6]. Previous efforts in the definition of the canine genome included combinations of gene identification and mapping [7], which was further improved by the release of a 7.6x draft sequence (Genome) [8]. Hence, the dog constitutes a unique resource for disease genetics.

A recently developed, normalized, canine retina-expressed sequence tag library [9], supported by an online database (DOG EST or DOG EST Project), currently catalogs 7,047 individual clones comprising more than 4,000 unique transcripts. About two-thirds of the clones in this library were identified as orthologous to annotated RefSeq sequences, and, of those, 41% corresponded, by sequence identity, to previously annotated canine cDNA entries. However, 1,418 of the transcripts remain to be annotated with certainty. In the present study, a subset of 553 clones was mapped using the well established canine/hamster hybrid panel RHDF_5000–2_ [10,11] to locate the corresponding retinal genes within the dog genome and, by comparative genomics, to infer their location in human.

Comparison of EST map positions obtained by RH and sequence mapping overcomes the disadvantages and limitations of each method alone, and offers the most reliable chromosomal locations for each EST. Such an integrated map provides substantial information on potential new candidate genes for heritable retinal disorders.

## Methods

### Clone identification and primer design

A set of 1,418 ESTs could not be identified with a high level of confidence by alignment with RefSeq genes or previously mapped canine cDNAs from the previously reported canine retinal EST library [9]. This particular set was then selected for mapping on the RH panel. A total of 35 EST sequences had to be excluded from primer design based on their respective sequences. Primer pairs for the remaining EST sequences were obtained in one of two ways. 227 were manually designed using the primer select option of the Lasergene™ software (Dnastar, Inc., Madison, WI). 1,156 were selected automatically using the batch primer design feature of GeneLooper™ (GeneHarbor, Inc., Rockville, MA) with an oligo Tm of 68 °C, and allowing primers within 100–150 bp of either end of the available sequence. From the latter 1,156 primer pairs, those resulting in PCR products smaller than 100 bp were excluded, and primers were sorted for increasing penalty scores assigned by the software; this yielded 920 primer pairs that were accepted for analysis. Together with the initial 227 pairs, a total of 1,147 EST sequences were thus assigned to the mapping project (Appendix 1).

### RH amplification and data collection

Each PCR product was amplified under standard conditions using 25 ng DNA template, 1 mM MgCl_2_, 0.2 mM of each dNTP, 0.2 µM of each respective primer, and 0.5 U Taq polymerase.

Following an initial denaturation step at 94 °C for 2 min, 35 cycles were performed at 94 °C for 15 s, 52 °C, 55 °C, 58 °C, or 62 °C, respectively (Appendix 1) for 15 s, and 72 °C for 40 s. This was then followed by a final extension at 72 °C for 5 min. All PCR products were visualized on 2% agarose gels.

In the first step, primer pairs were used to amplify canine genomic DNA. Those that failed to amplify a single product of the expected size were not continued in the analysis (result F, Appendix 1). Primers that reliably amplified canine genomic DNA (result D, Appendix 1) were then tested for differential amplification of dog and hamster DNA. Those that yielded either no detectable hamster amplicon or a product significantly different in size from the canine amplicon (result RH and M, Appendix 1) were subsequently used to amplify all 118 cell lines of the canine and hamster hybrid panel RHDF_5000–2_ together with previously used hamster and dog DNA as controls. PCR results were manually transferred into a computer file registering presence or absence of amplification for each cell line.

### Mapping algorithm

The data were analyzed against the previously published RHDF_5000–2_ map [12]. ESTs were assigned to chromosomes, based on the chromosomal location of the closest linked previously mapped RH marker [12], using the MultiMap [13] best-two-points function. Chromosomes for which marker density was too low to yield a consistent whole-chromosome linkage group were partitioned into blocks, and individual maps were created of each block. A framework map of each block was first created using MultiMap at a logarithm of the odds (LOD) of 3 or more and then filled in at continually lowering LOD scores. Chromosomes were oriented using markers previously placed on meiotic and RH maps [12].

### BLAST results and graphic display

Genomic localization for each EST was assigned for the current canine genome sequence (Genome Gateway; CanFam2, May 2005) using BLASTN (cutoff value *E*≤1*e^−100^*). The genomic position thus identified for each EST and selected anchor markers were displayed in a graph relative to the size of the respective chromosome. This was done using software developed at the Cornell University Computational Biology Service Unit (available from the authors upon request). RetNet (RetNet; status January 2009) was searched to identify the map location of hereditary retinal disorders on the human genome. For all such human disease loci mapped to intervals less than 25 Mb, the homologous region of the canine genome was established using BioMart, and refined according to the UCSC genome browser multiple alignments, disregarding alignments of less than 20 kb in length (Appendix 2).

## Results

### Performance of retinal ESTs on the RHDF_5000–2_ panel

**Figure 1:**
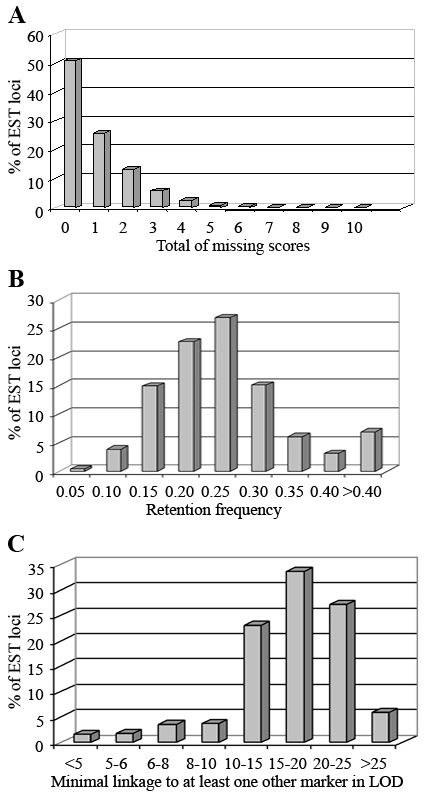
Quality control for retinal clones on the RHDF_5000–2_ panel. A total of 555 retinal clones were amplified from 118 cell lines representing the RHDF_5000–2_ panel. For each locus, we assessed both, the overall number of cell lines that could unambiguously be scored, and the number of cell lines amplifying the respective EST, for quality. Half of the loci were scored in each individual line with the balance of loci missing only few scores (**A**). The respective retention frequency resulting from amplification scores, on average, was 0.22 and showed a distribution that is similar to previously published data [12] using this panel (**B**). The good quality performance of EST amplification resulted in highly supported linkage to known markers (**C**) with most of the LOD scores above 10.

From the initial set of 1,418 ESTs with no detected homology to previously known sequences, amplification was attempted for a subset of 1,147 markers. Of these, 998 (87%) amplified a unique PCR product from canine genomic DNA without optimization, and 711 (62%) amplified a consistent and distinctive product from the RHDF_5000–2_ panel cell lines (Table 1). Roughly half of the ESTs tested could be scored satisfactorily for each of the 118 cell lines (Figure 1A). The overall presence of each EST marker on the panel (Figure 1B) was similar to previously published results (e.g., average retention frequency 22% in [12]). Furthermore, linkage to at least one other marker present in the RHDF5000–2 panel was found; this was supported by most two-point LOD scores higher than 10 (Figure 1C, Appendix 3).

**Table 1:** EST loci retained at each experimental step.

**EST loci**	**Number**	**Percent**
Tested	1,147	100.0
Amplified on dog DNA	998	87.0
Amplified satisfactorily on the RH5000–2 panel	711	62.0
Readable scores for all cell lines	555	48.4
Linked to at least one other marker	553	48.2
Mapped in unique position	501	43.7

To assess quality and efficiency of primer design, all primers were first amplified on dog DNA. Subsequently, working primer pairs were amplified on the RH panel, which proved to be the least efficient step. Of those satisfactorily amplifying on the panel, almost 80% could be scored in all cell lines, and only two of the scored ESTs did not provide sufficient linkage to at least on other marker. Finally, 90% of all linked markers were assigned to unique positions in the chromosome, thus yielding results for 44% of all tested primers without further optimization.

### Distribution of newly mapped retinal ESTs in the canine genome

Of 553 ESTs linked to known markers, 501 could be mapped to unique positions, ranging from 3 to 34 ESTs per chromosome (Table 2, Appendix 4). An additional 48 ESTs were linked, but not uniquely placed on the RH map. The remaining four EST loci collapsed with one of the other loci (Appendix 3) and were not counted as separate transcripts, reducing the total number of individually displayed loci to 549. The average number of ESTs per Mb mapped on the RH panel was calculated for each chromosome. These data were then compared to results obtained for all EST clones contained in the library (Table 2). With respect to the size of the chromosomes, relatively fewer clones were represented on the RH map for *Canis  familiaris* chromosome (CFA) 19, CFA32, and CFAX; clones represented in the complete library appeared to be evenly distributed throughout the genome with the exception of CFA19, which was synteny to portions of human chromosomes 2 and 4 [10]. It remains to be elucidated whether this is due to underrepresentation of retinal clones on CFA19 or, more likely, poorer annotation of this chromosome.

**Table 2:** ESTs and markers mapped per chromosome.

**CFA**	**CFA size, MB**	**blocks number**	**RH markers uniquely mapped**	**ESTs uniquely mapped**	**ESTs linked**	**EST/MB RH panel**	**ESTs database**	**EST/MB database**
1	137	4	135	34	1	0.26	895	6.53
2	99	2	90	20	0	0.20	733	7.40
3	105	4	85	18	4	0.21	580	5.52
4	100	2	93	16	0	0.16	578	5.78
5	99	3	108	26	0	0.26	599	6.05
6	87	2	79	16	1	0.20	462	5.31
7	94	4	114	15	0	0.16	673	7.16
8	86	1	76	13	3	0.19	689	8.01
9	77	4	95	16	3	0.25	522	6.78
10	80	3	60	15	3	0.23	702	8.78
11	86	1	96	14	2	0.19	592	6.88
12	85	2	113	20	1	0.25	564	6.64
13	75	1	54	8	1	0.12	331	4.41
14	72	1	75	9	1	0.14	559	7.76
15	75	1	76	16	0	0.21	497	6.63
16	73	3	64	18	2	0.27	368	5.04
17	80	2	80	17	1	0.23	435	5.44
18	66	1	79	16	1	0.26	543	8.23
19	66	3	57	4	0	0.06	212	3.21
20	66	1	93	13	5	0.27	507	7.68
21	61	4	86	8	1	0.15	408	6.69
22	61	1	53	7	6	0.21	393	6.44
23	61	1	51	7	2	0.15	318	5.21
24	73	1	51	16	0	0.22	369	5.05
25	60	2	68	14	1	0.25	451	7.52
26	48	1	50	9	1	0.21	389	8.10
27	57	1	67	15	1	0.28	532	9.33
28	55	1	53	19	1	0.36	310	5.64
29	51	1	53	8	0	0.16	313	6.14
30	47	2	42	15	2	0.36	415	8.83
31	50	2	34	8	2	0.20	265	5.30
32	51	1	29	3	0	0.06	447	8.76
33	41	1	39	11	0	0.27	215	5.24
34	50	1	41	6	1	0.14	221	4.42
35	38	1	24	5	0	0.13	187	4.92
36	41	1	44	4	1	0.12	220	5.37
37	40	1	47	6	0	0.15	214	5.35
38	38	2	24	5	0	0.13	149	3.92
X	139	3	52	8	0	0.06	927	6.67
Y	27	2	9	3	0	0.11	N/A	N/A

Each chromosome was mapped in individual linkage groups (blocks, column 3) containing previously mapped markers (column 4, reference [12]), ESTs mapped in unique positions (column 5), and ESTs linked, but not ordered on the chromosome (column 6). The number of RH-mapped ESTs per MB for each chromosome (column 7) was compared to the number of all ESTs currently in the database for each chromosome (column 8) per MB (column 9) to assess distribution of retinal expressed genes throughout the genome.

### Comparison of RH and sequence maps

For each chromosome, the linkage groups comprising all RH mapped retinal ESTs were aligned to the current canine genome sequence (Appendix 5). Apparent micro-rearrangements of markers among the maps were the most common discrepancy observed, particularly where markers could not be positioned on the RH map with high confidence. This is a familiar problem for markers located toward the end of a linkage group, e.g., CFA6 and CFA10 (Appendix 5).

Fourteen markers diverged in placement between the RH and sequence maps. Eight of these yielded best two-point linkage to a single chromosome, but could not be mapped in a unique position on the RH map (Appendix 5; e.g., DR010005B10H04 on CFA22). Therefore, correct genomic position of these ESTs should be assumed based on the sequence alignment, since it is the better supported method for these clones. The other six ESTs (DR01007A10C09 and DR010025B10A10 on CFA4, DR010009A20D03 on CFA6, DR010013A10E04 on CFA7, DR010027B10D03 on CFA17, and DR010015B20A07 on CFA19) were in complete disagreement with the RH map and the sequence assemblies (Appendix 5; no placements on D). Without additional information, correct genomic location of these ESTs cannot be determined.

### Indication of genome sequence alignment problems

A subset of ESTs proved difficult to align to a unique area of the sequence assembly. Despite the high cut-off value (*E*≤1*e^−100^*), 22 markers that were RH mapped to 16 different chromosomes showed significant sequence alignment to more than one chromosome. In each case, the multiple alignments included the chromosome assigned by the RH map. A consensus chromosomal position for these markers was thus based on the RH map (Appendix 5, e.g., DR010017A21D11 on CFA14, DR01005A20E07 on CFA15, placement on C, D, and E).

In addition to the aforementioned issues in placing individual markers, some genomic regions revealed consistent complications. Several markers RH mapped to CFA17 did not align to the same chromosome in the canine genome sequence draft; DR010027B10D03 mapped to CFA3, while DR010006A10A08, DR010025A10C12, DR010010B20B05, and DR010024A10E04 yielded multiple hits on CFA17, in addition to several alignments against alignments against a part of the sequence assembly not assigned to chromosomes (chrUN). Similarly, marker DR010026A20B10 on CFA11 aligned to multiple genomic locations. It is also worth mentioning that the three ESTs RH mapped to CFAY have significant sequence homology to locations on CFAX in the genome sequence assemblies.

### Identification of potential candidate genes for human retinal disorders

**Figure 2:**
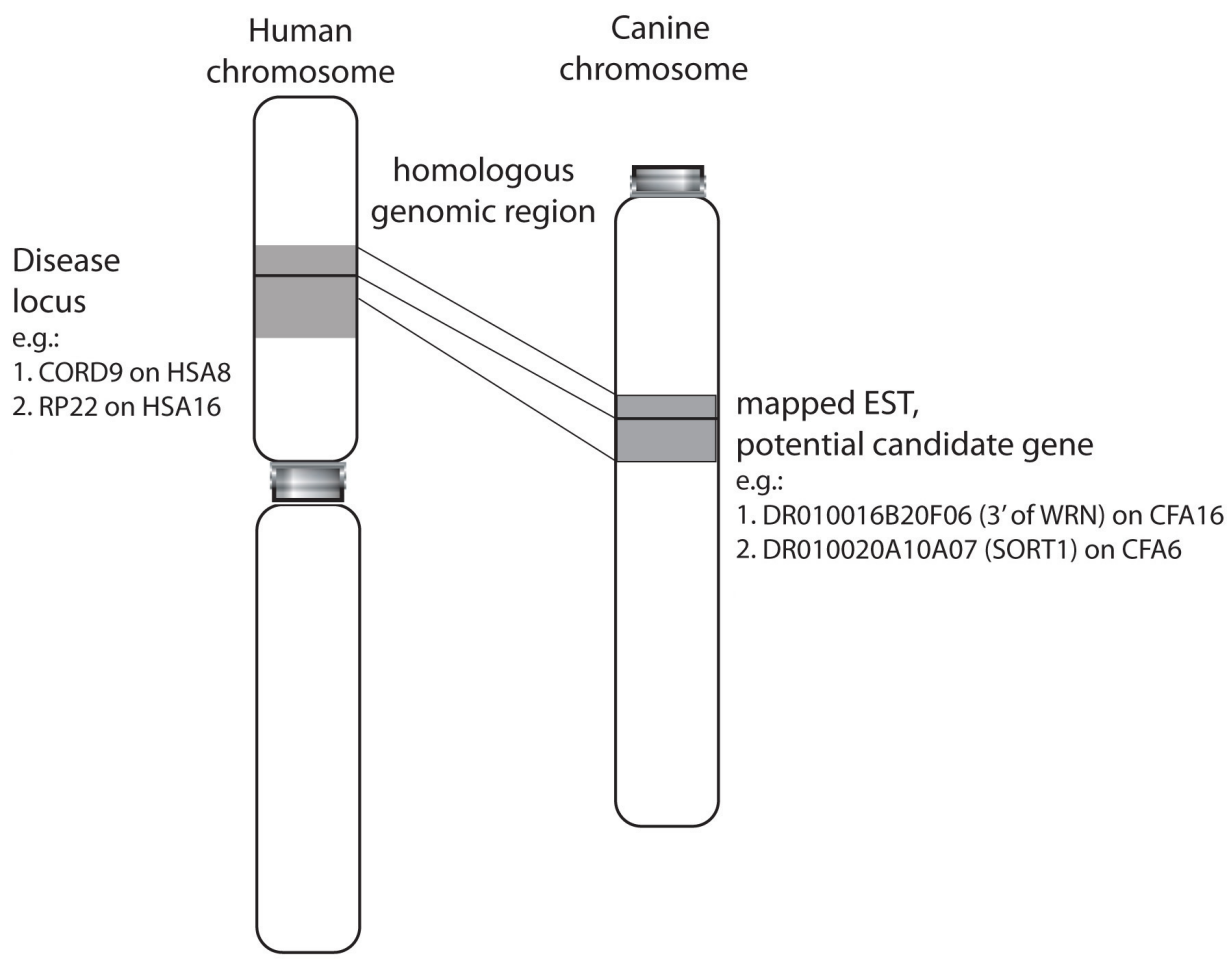
Identification of potential candidate genes for human retinal disease Human genomic intervals for known diseases (e.g., CORD9 on HSA8, RP22 on HSA16) were mapped against the canine genome to identify homologous regions, and EST within these regions of interest (e.g., DR010016B20F06 on CFA16, DR010020A10A07 on CFA6). A comprehensive list of these disorders and the number of corresponding ESTs contained within our library is given in Appendix 2. ESTs mapped in the presented research are also illustrated on the respective chromosomes in Appendix 5. Details on all clones can be obtained through a web database (DOG EST or DOG EST Project) to obtain insights into corresponding transcripts (e.g.: 1. WRN, 2. SORT1). We suggest that this tool provides new positional candidate genes for mapped human retinal disorders. This would allow for the identification of mutations in genes that are thus far unknown or have not yet been linked to retinal disorders, after the exclusion of conventional candidate genes.

The homologous canine genome locations for 28 mapped human retinal disease loci (RetNet), for which disease causing genes remain unknown, were identified on the canine sequence assembly (Figure 2, Appendix 2). On average, these identified homologous canine segments were 83% of the size of the corresponding human genome sequence interval, while the interval homologous to CORS2 (HSA11p12-q13.3) was covered by only 49%. The latter is likely because it spans the HSA11 centromere. Candidate ESTs were identified for these locations based on their positional annotation to identify potential candidate genes for the respective disorders. For only one locus, X-chromosomal RP24, no potential candidate clones mapped to the proposed homologous disease interval. For twenty of these comparative genome regions in the dog, which include clones placed on the presented RH maps (Appendix 2, shaded), the respective diseases intervals are also illustrated on the canine chromosome maps (Appendix 5).

## Discussion

### Comparison of mapping approaches

EST clones from the previously published canine retinal library [9] were re-aligned against RefSeq sequences, with a criterion cut-off value of *E*≤1*e^−3^* accepted as establishing homology. This yielded annotation for 80% of clones evaluated. In the present study, hypothesizing that the remaining, unidentified sequences might represent previously unrecognized retinal genes or control elements relevant to retinal function, 553 such retinal clones were mapped to the RHDF_5000–2_ panel with high confidence, and of these, 501 were mapped to unique positions (Table 1, Figure 1, and Appendix 3). The resulting RH map thus integrated these ESTs into the overall canine genome. Among the mapped ESTs, 159 sequences (29%) aligned to known RefSeq sequences, but the balance (71%; 394) remained unidentified.

Direct comparison of this integrated RH map to the assembled canine genome sequence became possible during the progress of the present project. This permitted independent confirmation of the location of mapped clones, comparative evaluation of the different mapping methods, and, as a consequence, overcame some of the limitations of each method. RH mapping is relatively unreliable toward the end of linkage groups, and does not provide highly reliable order for markers located close together. However, some of these problems have recently been addressed in a refined map for this panel [14]. Nonetheless, mapping of markers by sequence alignment is subject to errors in assembly of the underlying sequence. For example, gene duplications, pseudo genes, and other sequence similarities within a single genome can create both ambiguities in assembly, and multiple significant alignments for a single marker; this was recognized in 4% of the presented data. Furthermore, alignment of expressed sequences, which may be spliced, against a genome assembly, can be problematic since the spliced out sequence (e.g., introns) can cause difficulties in evaluating alignment scores.

The greater part of inconsistencies between placement on the RH and sequence maps were minor. These were more common at the ends of chromosomes rather than the middle. In particular, the multiple sequence alignments for ESTs mapping near the centromere of CFA11 and parts of CFA17 suggest sequence or assembly problems, or even potential genome duplications. In general, major disagreements between the compared maps were rare and resulted in only 1% of the chromosomal placement of ESTs to remain unresolved. While the data indicate room for improvement, these issues are likely to be resolved with the improvement of both RH maps and genome sequence assemblies. The synergistic combination of different approaches provides higher fidelity genome maps than does any single method alone [15].

### Genomic coverage of retinal ESTs

Overall, retinal ESTs analyzed in the current study do not appear to be concentrated in particular areas of the genome, but were distributed rather evenly throughout. There were, however, some departures from this general observation. CFA19, CFA32, and CFAX were relatively underrepresented by ESTs mapped in the present study, but only CFA19 showed the same underrepresentation when adjusted for all ESTs from the same library (Table 2). This suggests that the initial annotations were more efficient for CFA32 and CFAX and, thus, fewer clones were selected for additional mapping and annotation. For CFA19, however, in addition to a lower representation of retinal EST clones, one of the four ESTs RH-mapped to this chromosome aligned to a different chromosome (CFA27) in the canine genome sequence assembly, indicating a potential error in one or the other map.

We attempted to identify canine ESTs that potentially might represent novel candidate sequences for 28 mapped loci for hereditary human retinal disorders. Surprisingly, the only such locus that did not yield canine retinal ESTs contained neither in the complete EST library nor was presented in the RH mapped subset, was X-linked. This is especially remarkable considering the well recognized strong conservation of the mammalian X chromosome [16,17]. More importantly, all three ESTs mapped to CFAY on the RHDF_5000–2_ panel aligned close to the centromere of CFAX based on sequence homology. Based on the absence of CFAY sequences from the CanFam2 assembly, the correct assignment of the RH map linkage group to this chromosome cannot be verified by sequence alignments. One therefore is left to hypothesize whether this finding represent incorrect linkage in the RH map or functional transcripts are indeed encoded on CFAY. However, since the genomic CFAX sequence aligning to one of the clones, DR010006B10E05, contained a microsatellite that is not present in the EST, it is possible these transcripts have become inactivated on CFAX but are still functionally present on CFAY. Further studies will be necessary to confirm and interpret this potentially interesting finding.

### Candidate genes for human retinal disorders

Despite considerable progress in recent years, there has been a steady gap between identification of the mapped loci for heritable human retinal disorders, and characterization of the underlying gene and causative mutations (D graph). In part, this reflects an incomplete understanding of the genes critical for retinal development, function, and maintenance. Currently, 48 such loci are recognized for which no causative sequence change has been defined. For 20 loci, the candidate region is either not precisely defined or extends over more than 25 Mb. For the remaining 28 loci, we attempted to identify retina-expressed ESTs that might represent potential candidate genes, and were able to do so for all except the aforementioned X-linked RP24 locus. It should be acknowledged that some of these clones may be redundant—i.e., more than one EST might represent the same transcript. Thus, the number of potential retinal expressed candidate genes could be overestimated in our data set. Furthermore, genomic regions for some human disease loci also do not align unambiguously between human and dog, (e.g., MRST aligned to several areas on CFA3 and CFA30), and, consequently, we may not have covered the complete homologous disease intervals in the dog.

Confidence in the RH map location of an EST, and, concomitantly in its potential as a positional candidate, is strengthened when confirmed by sequence alignment with the corresponding genomic interval. This was achieved for the majority of ESTs (93.6%) in the current study. However, three clones potentially located within disease relevant areas were mapped to different genomic locations with the RH map when compared to the genomic sequence. DR010007A10C09, which aligns within the MRST homologous interval on CFA3 in the sequence assembly, was mapped with high confidence to CFA4 on the RH map (Appendix 5, CFA4, C). In contrast, DR010030A10D12 was located within the CORD9 interval on CFA16 in the RH map, but showed sequence homology just outside this interval in the sequence assembly; the same applies to DR010030A10G12 in the RP23 interval on CFAX. Despite these minor inconsistencies, however, homologous canine intervals have successfully been established for most of the human loci extracted from RetNet (Figure 2, Appendix 2).

The importance of correctly assigned location and tissue specificity of potential disease candidate genes has recently been demonstrated for progressive rod-cone degeneration (*prcd*) in the canine model. This disorder had previously been mapped to CFA9, and known retinal expressed genes within the disease critical interval had been excluded from causative association with disease [18-20]. Once the EST library was screened for positional candidates, transcripts expressing a novel gene were identified harboring the mutation responsible for *prcd* [21]. Subsequently, the gene was found to also cause RP in humans. It should be remarked that this effort was initially complicated by the fact that the disease critical interval was incorrectly assembled to CFA18 in the first pass of the canine genome assembly CanFam1. However, the canine draft sequence has been revised and is in high agreement with the presented RH mapping data.

A major advantage of comparative genomics lies in the validation of results and preliminary data in the absence of large patient cohorts to repeat or further narrow linkage data of known disease loci. One of the most recent genes identified to contribute to vision loss in humans is *PROM1* [22]. A missense mutation in the gene has been linked to formerly mapped loci MCDR2 and STGD4 in a screen of 12 positional candidate genes within the minimal overlapping disease interval of 12cM between markers D4S1582 and GATA1582G03. This region corresponds to 9.7 Mb (bp 65,599,776 −71,744,479; 90,673,048–94,195,452) located on CFA3 in the dog. The presented library contains 10 transcripts for this genomic location; the most abundant one of those, eight clones comprising contig 1265, represents the canine version of *PROM1*. Thus, a cross species comparison may have been able to assist in the ranking of candidate genes for this disease. With the high fidelity of genomic location established, this resource is well suited to assist with the investigation of known and new disease loci to speed up first steps in the identification and understanding of retinal disorders.

In conclusion, a comparative map of retinal genes allows identification of new candidate genes for retinal disorders in both dog and human, and provides a further step toward the complete categorization of genes relevant to retinal development and degeneration. Results of this study are integrated into the web database at DOG EST or DOG EST Project, and are publically accessible.

## Appendix 1.

To access the data, click or select the words “Appendix 1.” This will initiate the download of a (pdf) archive that contains the file. Amplification details and results (F=PCR failed, D=amplified from dog genomic DNA, RH=amplified on the RH panel, M=placed on the final map) for all ESTs tested.

## Appendix 2.

To access the data, click or select the words “Appendix 2.” This will initiate the download of a (pdf) archive that contains the file. Human retinal disease loci mapped against the canine genome. Based on the corresponding intervals, potential candidate genes were identified from the canine retinal EST library. The number of those ESTs is provided in the last column (EST contained in the complete database/subset al.so included in the currently presented RH mapping project). Genomic regions containing ESTs mapped in the present study (shaded) are also illustrated in Appendix 5.

## Appendix 3.

To access the data, click or select the words “Appendix 3.” This will initiate the download of a (pdf) archive that contains the file. Chromosomal location of each EST was assigned using the MultiMap [13] best-two points function using the previously published canine RH map [12]. Results are listed for each EST to demonstrate linkage to the closest marker (**A**) or indicate that the EST has collapsed with one of the markers and been placed in the same genomic location (**B**).

## Appendix 4.

To access the data, click or select the words “Appendix 4.” This will initiate the download of a (pdf) archive that contains the file. Markers were grouped into chromosomes according to results of the best-two points function (Appendix 3) and subsequently mapped within each chromosome using MultiMap [13]. Chromosomes for which marker density was too low to yield a consistent whole-chromosome linkage group were petitioned into blocks, and individual maps were created of each block. Final orientation and location of all markers on the chromosome are displayed according to previously established chromosome orientation [12]. Appendix lists all markers used for mapping, selected markers and EST locations are graphically displayed in Appendix 5.

## Appendix 5. Comparative maps of retinal expressed clones in the canine genome.

To access the data, click or select the words “Appendix 5.” This will initiate the download of a (pdf) archive that contains the file. Results of the EST mapping are graphically displayed for each chromosome indicating the relative position (**A**) of all mapped ESTs (**B**, blue) on the RH panel (**C**), the corresponding chromosome sequence draft (**D**), and potential alignments to nonhomologous chromosomes (**E**). Placement is indicated by horizontal lines, while oblique lines connect placements on different maps for the same EST. Chromosomes are oriented with centromeres located next to the respective legend on top of each page (**A**, 0); the artificial scale to the left (**A**) is proportional to the respective chromosome size based on the RH mapping. For orientation, selected markers are displayed on the RH map (**B**, red), and marker groups mapped in individual blocks are separated by black lines. ESTs linked, but not uniquely mapped to individual chromosome are displayed in *italic*. Intervals corresponding to mapped human disease loci (Appendix 2) are indicated on the right of each homologous region of the sequence drafts (**C**).
